# Association between migraine and depression, anxiety, and stress in the Aseer region: a cross-sectional study

**DOI:** 10.3389/fneur.2025.1650891

**Published:** 2025-08-26

**Authors:** Amjad Khalid Y. Abumilha, Abdullah Saeed A. Abukaftah, Mohammed Mushabab Al-Mudhi, Nojoud Ali Al Fareh, Abdulaziz Muflih Abudasser

**Affiliations:** ^1^Psychiatric Residents, Abha Mental Health Hospital, Abha, Saudi Arabia; ^2^Department of Psychiatry, Armed Forces Hospital Southern Region, Jeddah, Saudi Arabia; ^3^Department of Family Medicine, Aseer General Directorate of Health Affairs, Abha, Saudi Arabia; ^4^Department of Medicine, College of Medicine King Khalid University, Abha, Saudi Arabia; ^5^Department of Medicine, College of Medicine, King Khalid University, Abha, Saudi Arabia

**Keywords:** migraine, mental disorders, depression, stress, Saudi Arabia

## Abstract

**Aim:**

This study aimed to assess the association between migraine and depression, anxiety, and stress in the Aseer region of Saudi Arabia.

**Methods:**

A cross-sectional study was conducted in the Aseer region, targeting residents aged 18 years or older. The validity and reliability of the questionnaire were assessed through a pilot study and expert review. The final version of the questionnaire was distributed online to eligible participants.

**Results:**

A total of 395 eligible participants completed the study questionnaire. The ages of the participants ranged from 18 to over 40 years, with a mean age of 28.5 ± 12.1 years. A total of 159 (40.3%) participants had symptomatic migraine. Migraine increased the likelihood of having depression by approximately seven times (OR = 7.1; 95% CI: 3.9–12.7), anxiety disorder by approximately eight times (OR = 8.3; 95% CI: 4.4–15.6), and stress by approximately five times (OR = 5.2; 95% CI: 3.1–8.3), after adjusting for the effects of other personal characteristics.

**Conclusion:**

The study found that less than half of the participants met the clinical criteria for migraine, resulting in high levels of anxiety, depression, and stress, even after adjusting for other participant characteristics.

## Introduction

Migraine is a prevalent neurological condition and is recognized as the third leading cause of disability worldwide (1). It is characterized by recurring episodes of moderate to severe headaches, which are often accompanied by symptoms such as nausea, vomiting, and heightened sensitivity to light and sound (2). Women are more prone to experiencing migraines, which have been linked to psychological conditions such as stress, anxiety, and depression (3). In addition to increasing the disease burden for individuals, these comorbidities also have a substantial impact on society and healthcare systems (4). Similar to many other countries, Saudi Arabia has a high prevalence of migraine, affecting approximately 22–24% of the general population (5, 6).

Anxiety and depression are common symptoms among migraine patients. Individuals with migraine are two to five times more likely to experience these symptoms compared to those without migraine (7, 8). Studies have shown that approximately 25% of people with migraine also have depression, and up to 50% experience anxiety (9, 10).

Symptoms of anxiety and depression can affect various aspects of health. Individuals with migraine who also experience these symptoms tend to incur higher medical costs (11). They are also at a higher risk of suicide (12). Migraine patients who also experience anxiety or depression tend to have greater levels of disability than those without these mood disorders. Importantly, untreated mood disorders can reduce the effectiveness of headache treatments, and patients who do not receive appropriate care are less likely to follow recommended behavioral or medical therapies (12, 13). For these reasons, it is essential to ensure that patients with each of these conditions receive the best possible treatment.

In Saudi Arabia, there has been limited research on the relationship between migraine and psychiatric symptoms. Understanding this connection is crucial for developing targeted interventions and enhancing the quality of life for individuals with migraine. This study examined the association between migraine and levels of stress, anxiety, and depression in a Saudi Arabian population using a cross-sectional approach.

## Methodology

We conducted our study after obtaining ethical approval from the King Khalid University Committee of Research Ethics on 19 February 2023 (approval number ECM#2023-708A), in accordance with the Declaration of Helsinki.

Inclusion criteria: All available and accessible residents in the Aseer region aged 18 years or older were included.

Exclusion criteria: Individuals aged less than 18 years, those living outside the Aseer region, and those who refused to participate or did not complete the study questionnaire were excluded. Participants were recruited using a convenience sampling method from 29 February 2024to 21 July 2024. A pre-structured questionnaire was used as the data collection tool. The study questionnaire included the participants’ bio-demographic data, including age, sex, education, employment status, and psychiatric history. The second section included the Depression, Anxiety, and Stress Scale-21 (DASS-21) items for psychiatric comorbidities. The Arabic version of the DASS-21 was utilized to identify common mental disorders among secondary school children. The reliability of the Arabic DASS was examined in a previous study (14) and was found to be suitable (Cronbach’s alpha of 0.8). Depression was classified based on the scores obtained as follows: Normal (0–9), mild (10-13), moderate (14-20), severe (21-27), and extremely severe (≥ 28). Similarly, anxiety was classified as normal (0–7), mild (8-9), moderate (10-14), severe (15-19), and extremely severe (≥ 20). Likewise, stress was classified as normal (0–14), mild (15-18), moderate (19-25), severe (26-33), and extremely severe (≥ 34) (15). In addition, migraine was assessed using the Migraine Screen Questionnaire (MS-Q), which consists of five questions regarding the frequency and characteristics of headaches, as well as the presence or absence of migraine-related symptoms. A score of 0 was assigned for each negative answer (NO), and 1 was assigned for each positive answer (YES). A cutoff indicating suspicion of migraine was established at ≥ 4 points, while a score of <4 indicated no suspicion of migraine (16). The validity and reliability of the study questionnaire were assessed through a pilot study involving 25 participants and a review by experts. Modifications were made until the final study questionnaire was achieved. The tool’s reliability was 0.76, based on Cronbach’s alpha. The final version of the study questionnaire was distributed online via social media platforms to all eligible participants by the study researchers and their families until no new participants were recruited.

### Data analysis

The collected data were reviewed and entered into the Statistical Package for the Social Sciences (SPSS: IBM) (version 26) for analysis. All statistical analyses were conducted using two-tailed tests, with the alpha level set at 0.05. The results were considered significant if the *p*-value was less than or equal to 0.05. Descriptive statistics, including frequencies and percentages, were calculated for variables such as participants’ demographic characteristics, psychiatric history, DASS-21 domains (depression, anxiety, and stress), and items from the MS-Q. The overall levels of depression, anxiety, and stress and the prevalence of symptomatic migraine were visually presented. Cross-tabulations were created to display the distribution of mental health conditions and migraine according to demographic factors, using the Pearson chi-squared test or Fisher’s exact probability test when cell counts were low. The association between migraine and psychiatric comorbidities was examined using simple logistic regression for unadjusted analyses and hierarchical logistic regression for adjusted analyses, reporting adjusted odds ratios with 95% confidence intervals.

## Results

A total of 395 eligible individuals completed the study questionnaire. The participants’ ages ranged from 18 to over 40 years, with an average age of 28.5 ± 12.1 years. Of the participants, 214 (54.2%) were female and 383 (97%) were Saudi nationals. Regarding employment status, 137 (34.7%) were unemployed, 80 (20.3%) were students, 69 (17.5%) were employed in the government sector, 82 (20.8%) worked in the private sector, and 27 (6.8%) were retired. Exactly 200 participants (50.6%) were single, while 150 (38%) were married. In terms of educational attainment, 170 individuals (43%) had completed university education, 162 (41%) had a secondary education, and 37 (9.4%) held a postgraduate degree. The most commonly reported monthly income was between 5,000 and 15,000 Saudi Riyal (SAR) (56.2%), while 23.5% of the participants had a monthly income exceeding 15,000 SAR. Regarding mental health, 184 individuals (46.6%) reported no psychological disorders, whereas 73 participants (18.5%) had been diagnosed with both depression and anxiety (Table 1).

**Table 1:** Bio-demographic characteristics of the study participants, Aseer region, Saudi Arabia (*n* = 395).

Bio-demographic data	No	%
Age in years		
18–24	122	30.9%
25–30	138	34.9%
31–40	73	18.5%
> 40	62	15.7%
Sex		
Male	181	45.8%
Female	214	54.2%
Nationality		
Saudi	383	97.0%
Non-Saudi	12	3.0%
Employment		
Unemployed	137	34.7%
Student	80	20.3%
Governmental	69	17.5%
Private/military	82	20.8%
Retired	27	6.8%
Marital status		
Single	200	50.6%
Married	150	38.0%
Divorced/widow	45	11.4%
Educational level		
Below secondary	26	6.6%
Secondary	162	41.0%
University	170	43.0%
Post-graduate	37	9.4%
Monthly income		
< 5,000 SR	80	20.3%
5,000–15,000 SR	222	56.2%
> 15,000 SR	93	23.5%
Diagnosed with psychological disorders		
None	184	46.6%
Anxiety disorders	95	24.1%
Depression	43	10.9%
Both of them	73	18.5%

Table 2 Regarding depressive symptoms, 73.4% of the participants reported feeling that life was meaningless, 68.9% were unable to experience any positive feelings, 68.1% felt downhearted and blue, and 67.6% found it hard to summon the motivation to do things. For anxiety symptoms, 70.1% of the participants reported feeling scared without a clear reason. In addition, 69.4% noticed their heart beating even when they were not physically active, 67.8% experienced episodes of panic, and another 67.8% were aware of their heartbeat in the absence of exertion. Regarding stress symptoms, 76.5% of the participants reported difficulty unwinding, 71.6% had trouble relaxing, 68.6% felt irritable, and 68.6% experienced agitation.

**Table 2:** Depression, anxiety, and stress symptoms among the study participants based on the DASS-21.

DASS-21	Not at all		Little of the time		Sometimes		Most of the time	
	No	%	No	%	No	%	No	%
Depression								
I could not seem to experience any positive feeling at all	123	31.1%	113	28.6%	100	25.3%	59	14.9%
I found it difficult to work up the initiative to do things	128	32.4%	106	26.8%	90	22.8%	71	18.0%
I felt that I had nothing to look forward to	138	34.9%	108	27.3%	76	19.2%	73	18.5%
I felt down-hearted and blue	126	31.9%	108	27.3%	89	22.5%	72	18.2%
I was unable to become enthusiastic about anything	128	32.4%	127	32.2%	69	17.5%	71	18.0%
I felt I was not worth much as a person	129	32.7%	109	27.6%	91	23.0%	66	16.7%
I felt that life was meaningless	105	26.6%	121	30.6%	90	22.8%	79	20.0%
Anxiety								
I was aware of dryness in my mouth	152	38.5%	117	29.6%	83	21.0%	43	10.9%
I experienced breathing difficulty (e.g., excessively rapid breathing, breathlessness in the absence of physical exertion)	151	38.2%	120	30.4%	60	15.2%	64	16.2%
I experienced trembling (e.g., in the hands)	156	39.5%	111	28.1%	64	16.2%	64	16.2%
I was worried about situations in which I might panic and make a fool of myself	127	32.2%	118	29.9%	67	17.0%	83	21.0%
I felt I was close to panicking	127	32.2%	104	26.3%	88	22.3%	76	19.2%
I was aware of the action of my heart in the absence of physical exertion	121	30.6%	118	29.9%	89	22.5%	67	17.0%
I felt scared without any good reason	118	29.9%	106	26.8%	85	21.5%	86	21.8%
Stress								
I found it hard to wind down	93	23.5%	138	34.9%	85	21.5%	79	20.0%
I tended to overreact to situations	129	32.7%	116	29.4%	76	19.2%	74	18.7%
I felt that I was using a lot of nervous energy	129	32.7%	103	26.1%	74	18.7%	89	22.5%
I found myself getting agitated	124	31.4%	105	26.6%	91	23.0%	75	19.0%
I found it difficult to relax	112	28.4%	114	28.9%	83	21.0%	86	21.8%
I was intolerant of anything that kept me from getting on with what I was doing	145	36.7%	101	25.6%	84	21.3%	65	16.5%
I felt that I was rather touchy	124	31.4%	120	30.4%	74	18.7%	77	19.5%

**Figure 1:**
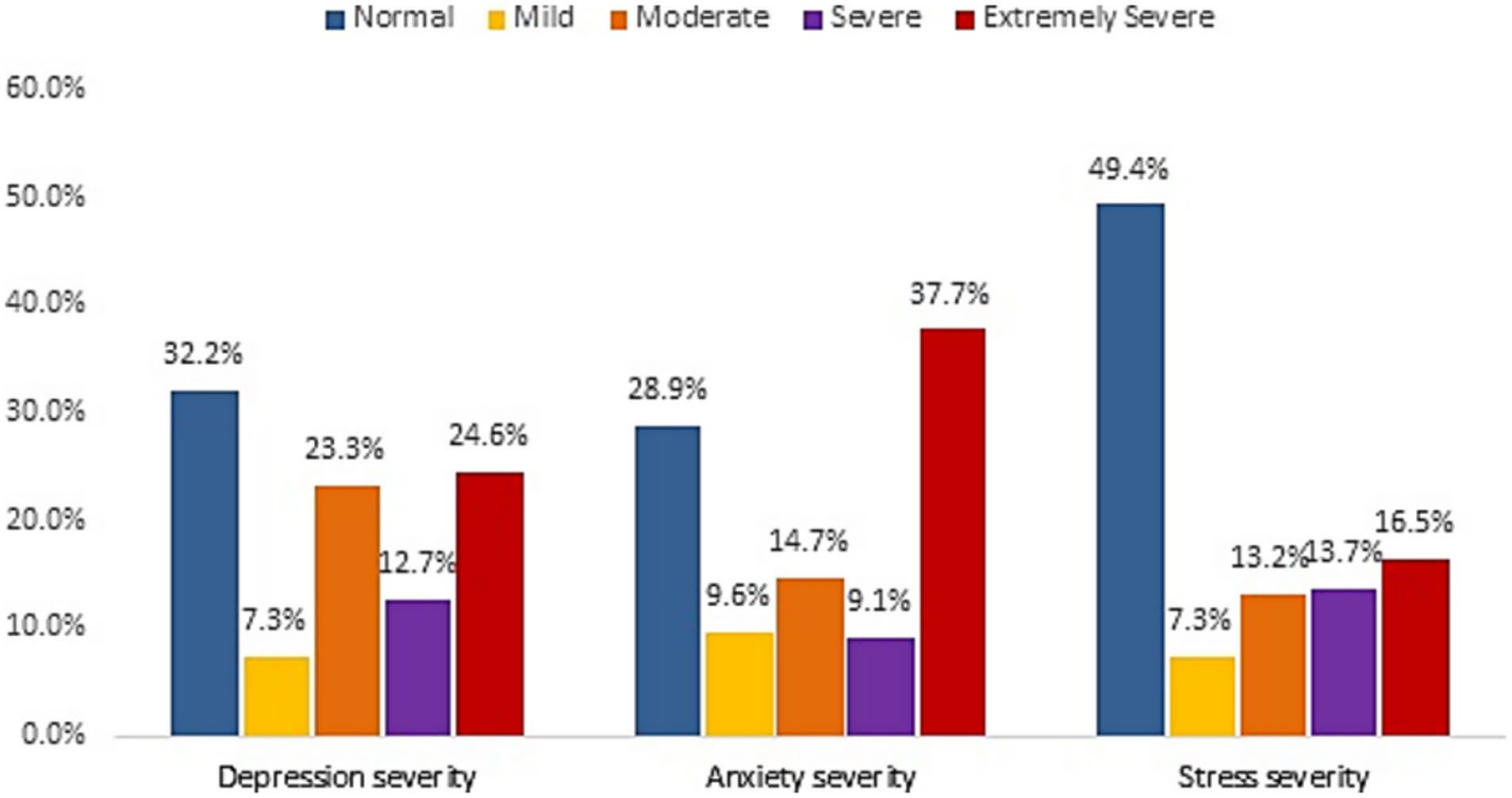
Depression, stress, and anxiety levels among the participants.

Figure 1 a total of 67.8% of the participants exhibited significant symptoms of depression, with 37.3% experiencing severe or extremely severe levels. Significant anxiety symptoms were identified in 71.1% of the participants, and 44.8% of these cases were classified as severe or extremely severe. Similarly, 50.6% of the participants had notable stress symptoms, with 30.2% reporting severe or extremely severe stress.

Table 3 a total of 113 participants (28.6%) were diagnosed with migraine. Frequent or intense headaches were reported by 200 individuals (50.6%), and 128 (32.4%) experienced headaches lasting more than 4 h. Nausea accompanying headaches was noted in 203 participants (51.4%), while 214 (54.2%) reported sensitivity to light or noise during headaches. In addition, 195 individuals (49.4%) stated that headaches limited their physical or intellectual activities. Overall, 159 participants (40.3%) met the criteria for symptomatic migraine (Figure 1).

**Table 3:** Migraine headache assessment among the study participants, Aseer region, Saudi Arabia.

MS-Q	Yes		No	
	No	%	No	%
Previously diagnosed with migraine	113	28.6%	282	71.4%
Do you have frequent or intense headaches?	200	50.6%	195	49.4%
Does your headache last more than 4 h?	128	32.4%	267	67.6%
Do you usually experience nausea when you have a headache?	203	51.4%	192	48.6%
Does light or noise bother you when you have a headache?	214	54.2%	181	45.8%
Do headaches limit any of your physical or intellectual activities?	195	49.4%	200	50.6%

Table 4 a total of 54.8% of the participants over 40 years had migraine, compared to 29.5% of those aged 18–24 years, a difference that was statistically significant (*p* = 0.008). Symptomatic migraine was observed in 59.3% of the retired individuals compared to 27.5% of the students, with the difference being statistically significant (*p* = 0.001). Asymptomatic migraine was identified in 59.3% of the retired participants, whereas only 27.5% of the students experienced it, and this difference was statistically significant (*p* = 0.001).

**Table 4:** Factors associated with migraine headache among the study participants.

Factors	Have symptomatic migraine?				*P*-value
	Yes		No		
	No	%	No	%	
Age in years					
18-24	36	29.5%	86	70.5%	0.008*
25–30	57	41.3%	81	58.7%	
31–40	32	43.8%	41	56.2%	
> 40	34	54.8%	28	45.2%	
Sex					
Male	67	37.0%	114	63.0%	0.228
Female	92	43.0%	122	57.0%	
Nationality					
Saudi	157	41.0%	226	59.0%	0.091^
Non-Saudi	2	16.7%	10	83.3%	
Employment					
Unemployed	67	48.9%	70	51.1%	0.001*
Student	22	27.5%	58	72.5%	
Governmental	32	46.4%	37	53.6%	
Private/military	22	26.8%	60	73.2%	
Retired	16	59.3%	11	40.7%	
Marital status					
Single	70	35.0%	130	65.0%	0.030*
Married	64	42.7%	86	57.3%	
Divorced/widow	25	55.6%	20	44.4%	
Educational level					
Below secondary	7	26.9%	19	73.1%	0.001*
Secondary	51	31.5%	111	68.5%	
University	78	45.9%	92	54.1%	
Post-graduate	23	62.2%	14	37.8%	
Monthly income					
< 5,000 SR	32	40.0%	48	60.0%	0.101
5,000–15,000 SR	81	36.5%	141	63.5%	
> 15,000 SR	46	49.5%	47	50.5%	

P: Pearson X^2^ test, ^: Exact probability test. *p < 0.05 (significant).

Table 5 among the study participants, 80.8% of the female individuals experienced at least one psychiatric comorbidity, compared to 65.7% of the male individuals (*p* = 0.001). Similarly, 85.2% of the retired individuals had at least one psychiatric comorbidity, compared to 65% of the students (*p* = 0.048). In addition, psychiatric comorbidities were present in 78.8% of the university graduates, compared to 66% of the individuals with only secondary education (*p* = 0.026).

**Table 5:** Factors associated with psychiatric comorbidities among the study participants.

Factors	Psychiatric comorbidities				*P*-value
	Yes		No		
	No	%	No	%	
Age in years					
18–24	83	68.0%	39	32.0%	0.183^
25–30	107	77.5%	31	22.5%	
31–40	52	71.2%	21	28.8%	
> 40	50	80.6%	12	19.4%	
Sex					
Male	119	65.7%	62	34.3%	0.001*
Female	173	80.8%	41	19.2%	
Nationality					
Saudi	282	73.6%	101	26.4%	0.451^
Non-Saudi	10	83.3%	2	16.7%	
Employment					
Unemployed	109	79.6%	28	20.4%	0.048*
Student	52	65.0%	28	35.0%	
Governmental	52	75.4%	17	24.6%	
Private/military	56	68.3%	26	31.7%	
Retired	23	85.2%	4	14.8%	
Marital status					
Single	143	71.5%	57	28.5%	0.524
Married	114	76.0%	36	24.0%	
Divorced/widow	35	77.8%	10	22.2%	
Educational level					
Below secondary	22	84.6%	4	15.4%	0.026*
Secondary	107	66.0%	55	34.0%	
University	134	78.8%	36	21.2%	
Post-graduate	29	78.4%	8	21.6%	
Monthly income					
< 5,000 SR	53	66.3%	27	33.8%	0.171
5,000–15,000 SR	166	74.8%	56	25.2%	
> 15,000 SR	73	78.5%	20	21.5%	

P: Pearson X^2^ test, ^: Exact probability test. *P < 0.05 (significant).

Table 6 severe or extremely severe depression was present in 61.6% of the participants with migraine, compared to 20.8% of those without migraine (*p* = 0.001). Similarly, 69.8% of the individuals with migraine experienced severe or extremely severe anxiety, compared to 31.3% of the non-migraine participants (*p* = 0.001). Severe or extremely severe stress was observed in 54.4% of the patients with migraine, compared to 14.4% of those without migraine (*p* = 0.001). Overall, after controlling for other personal factors, migraine was associated with approximately a sevenfold increase in the likelihood of depression (OR = 7.1; 95% CI: 3.9–12.7), an eightfold increase in the likelihood of anxiety disorders (OR = 8.3; 95% CI: 4.4–15.6), and a fivefold increase in the likelihood of stress (OR = 5.2; 95% CI: 3.1–8.3).

**Table 6:** Crude and adjusted effects of migraine on the study participants’ psychiatric statuses (depression, anxiety, and stress).

Psychiatric co-morbidities	Have symptomatic migraine?				*P*-value	OR_c_ (95% CI)	OR_A_ (95% CI)
	Yes		No				
	No	%	No	%			
Depression severity							
Normal	17	10.7%	110	46.6%	0.001*		
Mild	6	3.8%	23	9.7%		7.3 (4.2–12.8) *	7.1 (3.9–12.7) *
Moderate	38	23.9%	54	22.9%			
Severe	21	13.2%	29	12.3%			
Extremely severe	77	48.4%	20	8.5%			
Anxiety severity							
Normal	14	8.8%	100	42.4%	0.001*		
Mild	7	4.4%	31	13.1%		7.6 (4.1–13.9) *	8.3 (4.4–15.6) *
Moderate	27	17.0%	31	13.1%			
Severe	13	8.2%	23	9.7%			
Extremely severe	98	61.6%	51	21.6%			
Stress severity							
Normal	44	27.7%	151	64.0%	0.001*		
Mild	10	6.3%	19	8.1%		4.6 (2.9–7.1) *	5.2 (3.1–8.3) *
Moderate	20	12.6%	32	13.6%			
Severe	29	18.2%	25	10.6%			
Extremely severe	56	35.2%	9	3.8%			

OR_C_: Crude odds ratio OR_A_: Adjusted odds ratio for personal characteristics CI: Confidence interval. *p < 0.05 (significant).

## Discussion

This study investigated the relationship between migraine and levels of depression, anxiety, and stress among individuals living in the Aseer Region. Research has consistently shown a strong two-way relationship between migraine and psychiatric disorders (17). Migraine is a disabling disorder that adversely affects physical health, overall well-being, relationships with family, and performance at work or school. Additionally, it creates a considerable financial burden. Migraine often leads to significant challenges in psychological health and reduces the overall quality of life. (18).

In this study, symptomatic migraine was identified in 40.3% of the participants, with notably higher rates observed among older adults, individuals who were divorced, and those with higher levels of education. A much higher prevalence was reported by Almalki et al. (19) in Taif, where approximately 90% of participants reported migraine attacks. A systematic review revealed that migraine prevalence among the general population in Arab countries ranged between 2.6 and 32%, which is lower than the prevalence estimated in the current study. This variability in reported prevalence may be due to differences in the assessment tools and definitions used for migraine. Other studies have reported lower prevalence rates, such as headache occurring in 53.2% of individuals in Brazil (20), 33.8% in Nairobi (21), and 27.9% in Kuwait (22).

Regarding psychiatric comorbidities, the present study found that approximately two-thirds of the participants exhibited significant symptoms of depression, with 37.3% of them experiencing severe or extremely severe depression. In addition, over two-thirds of the participants experienced notable anxiety symptoms, with fewer than half of these cases classified as severe or extremely severe. Similarly, significant stress symptoms were reported by 50.6% of the participants, indicating that approximately half of the study group experienced stress.

A large Polish study conducted by Waliszewska-Prosol et al. (23), which included 3,225 migraine patients, indicated that approximately one-fifth of the patients were diagnosed with depression and anxiety using the Patient Health Questionnaire-9 (PHQ-9). In addition, a large multicenter study by Rosignoli et al. (24) demonstrated that 12 and 17% of migraine patients had anxiety and depression, respectively, as diagnosed using the ICD-10 code. A Turkish study by Semiz et al. (25), which evaluated psychiatric comorbidities among migraine patients using structured clinical interviews, reported that only 23.1% had a current psychiatric diagnosis and 43.2% had a lifetime diagnosis. Similarly, in a large-scale US population-based study by Jette et al. (26), migraine patients had approximately twice the prevalence of disorders such as major depression, panic disorder, bipolar disorder, and social phobia compared to non-migraine controls. Furthermore, in an outpatient cohort from Taiwan described by Yin et al. (37), only 6% of migraine patients were diagnosed with depression, while 16.5% were diagnosed with anxiety. Altwaijri et al. (27) reported that 40.1% of Saudi adults had a mental disorder, with anxiety disorders present in 26.8% of participants, disruptive behavior disorders in 15.4%, mood disorders in 9.6%, substance use disorders in 4%, and eating disorders in 7.0%. These rates are considerably lower than the prevalence found in the current study. According to the Saudi National Mental Health Survey, 12% of the Saudi population were diagnosed with anxiety disorders, 6% reported major depressive disorder, and 1.9% had generalized anxiety disorder. Research indicates that global prevalence rates are 28.0% for depression, 26.9% for anxiety, 24.1% for post-traumatic stress symptoms, 36.5% for stress, 50.0% for psychological distress, and 27.6% for sleep disturbances, which are comparable to the findings in the current study (28).

The present study found that individuals with migraine had significantly higher rates and greater severity of depression, anxiety, and stress compared to those without migraine. On average, migraine increased the risk of developing depression, anxiety, and stress by approximately sixfold—most notably anxiety (eightfold) and least for stress (fivefold). Similarly, Hamelsky SW and Lipton (29) reported that adults with migraine frequently experience anxiety and depression; they are two to four times more likely to experience depression and anxiety compared to adults in the general population. Alwhaibi M et al. (30) found that among individuals with migraine, 11.2% experienced depression, 14.6% had anxiety, and 13.7% had both conditions—rates that are considerably lower than those observed in the current study. A study in Makkah revealed that migraine cases had high levels of stress (63.9%), anxiety (63.6%), and depression (55%) (31). In the Aseer region, AlQarni MA et al. (32) found that depression was present in 26.1% of migraine patients, compared to 10.9 and 6.6% in NMH cases and adults with no headaches, respectively. Duan S et al. (33) found that individuals with anxiety had an adjusted odds ratio of 3.1 (95% CI: 1.387–7.141) and those with depression had an adjusted odds ratio of 5.1 (95% CI: 1.755–15.322) for developing migraine, indicating a significantly increased risk. Several studies have found that individuals with migraine experience depression and anxiety disorders at rates 2–10 times higher than those observed in the general population (34, 35). These results confirm a strong association between migraine and an increased risk of depression, anxiety, and stress, aligning with the findings of previous studies.

Recent literature highlights the potential benefits and complexities of combining prophylactic treatments for migraine. As summarized by Pellesi et al. (36), combining conventional oral prophylactics (e.g., beta-blockers, topiramate, and amitriptyline) with more advanced options, such as onabotulinumtoxinA, calcitonin gene-related peptide (CGRP)-targeted monoclonal antibodies, or gepants, may enhance the efficacy by targeting multiple pathophysiological pathways simultaneously.

However, this polytherapy approach presents practical challenges. High cost, limited access, and possible major side effects are significant barriers to using combinations such as CGRP monoclonal antibodies with onabotulinumtoxinA or dual CGRP modalities (36). Moreover, special consideration is needed for patients with comorbid conditions. A comprehensive evaluation of efficacy, safety, drug–drug interactions, and patient-specific factors is essential before initiating combination prophylaxis.

## Limitations

This study has several limitations that should be considered when interpreting the findings. First, the sex distribution in our sample was nearly equal, which does not reflect the well-established higher prevalence of migraine among female individuals in the general population. This may have contributed to a lower-than-expected overall migraine prevalence and could affect the generalizability of the results in female-dominated migraine populations. Second, the study relied entirely on self-reported screening tools to identify migraine symptoms and psychiatric comorbidities. These instruments, although validated, do not replace formal clinical diagnosis, and their reliance on subjective self-reporting may introduce recall or response bias. Third, no clinical verification was conducted for migraine diagnosis, psychiatric disorders, or medication use. Participants with other chronic illnesses or those taking medications that could influence thier mood or sleep were not excluded, which may have confounded some of the associations observed. Fourth, the cross-sectional design prevents causal inference. Fifth, although a considerable number of participants in our study were retirees, we did not screen for their prior occupational experiences, including migraine-related absenteeism or presenteeism. This limits our ability to assess whether migraine contributed to work-related disability or early retirement, which is a known concern in chronic migraine populations. Future research should explore these dimensions to better understand the long-term socioeconomic burden of migraine. Finally, sociocultural factors—including stigma around mental illness, religious coping behaviors, and regional stressors—were not systematically assessed in this study; however, they may influence the expression, reporting, and management of both migraine and psychological distress. Future studies should incorporate these contextual factors to improve interpretation and relevance.

## Conclusion and recommendations

The present study found that fewer than half of the participants met the clinical criteria for migraine, accompanied by elevated levels of anxiety and depression and a lower prevalence of stress. After controlling for other participant characteristics, migraine was significantly linked to an increased likelihood of anxiety, depression, and stress. Accurate identification and effective management of migraine can help lower the psychological impact on affected individuals. These findings highlight the importance of public health strategies that promote lifestyle changes to substantially decrease both mental health issues and migraine episodes.

## Data Availability

The raw data supporting the conclusions of this article will be made available by the authors, without undue reservation.
